# De Novo Transcriptome Analysis Reveals Putative Genes Involved in Anthraquinone Biosynthesis in *Rubia yunnanensis*

**DOI:** 10.3390/genes13030521

**Published:** 2022-03-16

**Authors:** Rongfei Zhang, Yuanyuan Miao, Lingyun Chen, Shanyong Yi, Ninghua Tan

**Affiliations:** 1 Department of TCMs Pharmaceuticals, School of Traditional Chinese Pharmacy, China Pharmaceutical University, Nanjing 211198, China; zrf1327182884@126.com (R.Z.); miaoyuanyuan1989@163.com (Y.M.); lychen@cpu.edu.cn (L.C.); 2 Department of Biological and Pharmaceutical Engineering, West Anhui University, Lu’an 237012, China

**Keywords:** *Rubia yunnanensis* Diels, RNA-sequencing, transcriptome, anthraquinone biosynthesis, gene expression

## Abstract

*Rubia yunnanensis* Diels (*R. yunnanensis*), a Chinese perennial plant, is well-known for its medicinal values such as rheumatism, contusion, and anemia. It is rich in bioactive anthraquinones, but the biosynthetic pathways of anthraquinones in *R. yunnanensis* remain unknown. To investigate genes involved in anthraquinone biosynthesis in *R. yunnanensis*, we generated a de novo transcriptome of *R. yunnanensis* using the Illumina HiSeq 2500 sequencing platform. A total of 636,198 transcripts were obtained, in which 140,078 transcripts were successfully annotated. A differential gene expression analysis identified 15 putative genes involved in anthraquinone biosynthesis. Additionally, the hairy roots of *R. yunnanensis* were treated with 200 µM Methyl Jasmonate (MeJA). The contents of six bioactive anthraquinones and gene expression levels of 15 putative genes were measured using ultra performance liquid chromatography coupled with mass spectrometry (UPLC-MS/MS) and real-time quantitative polymerase chain reaction (RT-qPCR), respectively. The results showed that the expressions levels for 11 of the 15 genes and the contents of two of six anthraquinones significantly increased by MeJA treatment. Pearson’s correlation analyses indicated that the expressions of 4 of the 15 putative genes were positively correlated with the contents of rubiquinone (Q3) and rubiquinone-3-*O*-*β*-d-xylopranosyl-(1→6)-*β*-d-glucopyranoside (Q20). This study reported the first de novo transcriptome of *R. yunnanensis* and shed light on the anthraquinone biosynthesis and genetic information for *R. yunnanensis*.

## 1. Introduction

*Rubia yunnanensis* Diels, a perennial herb in the family of Rubiaceae, distributes at 1700–2500 m altitude in Yunnan and Sichuan provinces, Southwest China [1]. Its roots and rhizomes, known as “XiaoHongShen”, have been used as one of Chinese traditional medicines (TCMs) for treating rheumatism, contusion, menoxenia, tuberculosis, and anemia [2]. This species is used as a local alternative of the TCMs *Rubia cordifolia* [3]. Recent studies showed that *R. yunnanensis* has a wide range of bioactivities, such as antihyperlipidemic [4], antitumor [5], antiplatelet aggregation [6], antibacterial, and antifungal [7] properties. These properties could be attributed to these active quinones including anthraquinones and naphthoquinones derivatives, i.e., rubiquinone (Q3), 3-hydroxy-2-hydroxymethyl-9,10-anthraquinone (Q4), rubiquinone-3-*O*-(4′-*O*-acetyl)-*α*-l-rhamnopyranosyl-(1→2)-*β*-d-glucopyranoside (Q19), rubiquinone-3-*O*-*β*-d-xylopranosyl-(1→6)-*β*-d-glucopyranoside (Q20), mollugin (Q17), and 1′-methoxy-2′-hydroxy-9,10-dihydromullugin (Q18) (Supplementary Figure S1) [8,9]. Due to its medicinal values, the demand for *R. yunnanensis* keeps increasing. However, knowledge about the genetics of *R. yunnanensis* is limited, which has hindered the investigation of metabolites synthesis.

Anthraquinones, constituting a class of the parent structure 9,10-dioxoanthracene, are synthesized by bacteria [10], fungi [11], insects [12], and plants [13]. Natural anthraquinones not only possess medicinal benefits but also possess values in pigments, foods, and cosmetics [14,15]. For instance, the roots and rhizomes of *Rubia*, which are generally rich in anthraquinones, are used as natural dyestuffs for clothes and foods in industry. Two main biosynthesis pathways for the skeleton of anthraquinones have been reported: (1) the polyketide pathway in Leguminosae, Rhamnaceae, and Caesalpiniaceae and (2) a combination of the shikimate, mevalonic acid (MVA), and 2-methyl-erythritol 4-phosphate (MEP) pathways in Rubiaceae [16,17]. The latter pathways comprise three benzene rings, A, B, and C. The rings A and B are derived from 1,4-dihydroxy-2-naphthoic acid via the shikimate pathway [18], and the ring C is derived from isopentenyl diphosphate (IPP)/3, 3-dimethylallyl diphosphate (DMAPP) via the MVA/MEP pathway [19]. Although we identified four glycosyltransferase genes involved in the biosynthesis of anthraquinone glycosides in *R. yunnanensis* [20], no other enzymes in anthraquinone biosynthesis have been fully identified. Moreover, the biosynthesis pathway of anthraquinones in *R*. *yunnanensis* remains unknown.

With advances in sequencing technologies, the Illumina HiSeq as a cost-efficient RNA-sequencing (RNA-seq) has become an effective tool to obtain candidate genes involved in the biosynthesis of active substances in non-model plants [21], such as tanshinones and salvianolic acids (*Salvia miltiorrhiza*) [22], C19-diterpenoid alkaloids (*Aconitum carmichaelii*) [23], anthraquinones (*Aloe vera*) [24], and flavonoids (*Gentiana straminea*) [25]. The MeJA induction has been established as an ideal model in plants for studying biosynthetic pathways of various classes of active secondary metabolites [26], such as ginsenoside [27], tanshinone [28], abietane diterpenes [29], and so on. After MeJA treatment, candidate genes that are essential for the secondary metabolite synthetic pathways can be easily captured by sequencing technologies and metabolic analysis. Combined with MeJA treatment, the RNA-seq technology can be used to study the molecular mechanism of anthraquinone biosynthesis in *R. yunnanensis* [30].

To investigate the genes involved in anthraquinones synthesis, we here generated a de novo transcriptome of *R. yunnanensis* using the Illumina HiSeq platform. The assembled transcripts were functionally annotated, and the genes related to anthraquinone biosynthesis were analyzed by differential gene expression analyses. The hairy root culture of *R. yunnanensis* with high production of quinones [31] was treated with MeJA for correlation analysis between the expressions of the putative genes and anthraquinone contents.

## 2. Materials and Methods

### 2.1. Sample Preparation

The seeds of *R.yunnanensis* were collected from Kunming, Yunnan Province, China in October 2016. Seeds were grown in 25 × 20 cm pots in a greenhouse with a condition of 25 °C, light for 16 h (intensity of illumination at a constant 30,000 Lu×), and dark for 8 h. The roots and a mixture of stems and leaves of one-year-old *R. yunnanensis* were collected and immediately frozen at −80 °C.

Hairy roots of *R. yunnanensis* established by our previous work [31] were cultured in 250 mL of 1/2MS liquid medium at pH 5.8, 25 °C, and in dark. After 30 days of cultivation, 200 μM MeJA dissolved in ethanol was added as the elicitation. The hairy roots were harvested at 0, 1, 6, 12, and 24 h after treatment. For the control groups, the elicitation was replaced by ethanol at the same volume. All the treatments were performed with three biological replicates.

### 2.2. RNA Isolation and cDNA Library Preparation for Illumina Sequencing

Total RNA was separately isolated from *R. yunnanensis* roots and a mixture of stems and leaves using EASY spin Plus Complex Plant RNA kit (RN53, Aidlab Biotechnologies Co., Ltd., Beijing, China) according to the manufacturer’s instructions. Each sample has three replicates. RNA quality was evaluated using a Nanodrop 2000 and Agilent 2100 (Supplementary Table S1). After treating with RNase-free DNase I (Katara, Dalian, China) for 30 min at 37 °C to remove the residual DNA, mRNA was enriched using mRNA Capture Beads. The fragmentation buffer was added to interrupt the mRNA to short fragments. SMART^TM^ cDNA Synthesis Kit (Clontech, Mountain View, CA, USA) was used to synthesize the single-stranded cDNA with the short fragments as templates. Subsequently, double-stranded cDNA was synthesized using the Advantage II Polymerase Kit (Clontech, Mountain View, CA, USA) including RNase H, dNTPs, and DNA polymerase I. The products were purified with EasyPure PCR Purification Kit (TransGen Biotech, Beijing, China), followed by adding poly(A). Finally, the library was sequenced using Illumina HiSeq™2500 with a paired-end (PE) sequencing strategy.

### 2.3. De Novo Assembly and Sequence Analysis

Raw reads were filtered by SOAPnuke (https://github.com/BGI-flexlab/SOAPnuke/, accessed on 10 February 2018) [32]. De novo assembly was carried out using Trinity v2.40. Assembled transcripts were then clustered to remove redundancies using the CDHIT (http://weizhongli-lab.org/cd-hit/, accessed on 10 February 2018) [33].

### 2.4. Functional Annotation of Transcripts

To obtain annotation, transcripts were compared with public databases using NCBI BLASTX [34]. The public databases included NCBI nucleotide sequences (NT) (http://www.ncbi.nlm.nih.gov/, accessed on 10 February 2018), NCBI non-redundant protein sequences (NR) (http://www.ncbi.nlm.nih.gov, accessed on 10 February 2018), Swiss-Prot (http://www.ebi.ac.uk/uniprot/, accessed on 10 February 2018), Protein family (Pfam) (http://www.ncbi.nlm.nih.gov/, accessed on 10 February 2018), euKaryotic Ortholog Groups (KOG) (http://www.ncbi.nlm.nih.gov/, accessed on 10 February 2018), Kyoto Encyclopedia of Genes and Genomes (KEGG) (http://www.genome.jp/kegg/, accessed on 10 February 2018), and Gene Ontology (GO) (http://www.genome.jp/kegg, accessed on 10 February 2018) databases.

### 2.5. Identification of Differentially Expressed Genes (DEGs)

Clean reads of each sample were mapped to the assembled transcripts with Bowtie2 [35]. Then, the FPKM (fragments per kilobase of per million mapped reads) for each transcript was calculated using RSEM software [36]. The FPKM quantified the expression abundance of transcripts in each sample. Last, edgeR software [37] was used to perform differential gene expression analyses based on the FPKM. When false discovery rate (FDR) ≤0.05 and an absolute value of the log2 ratio > 1.5, the transcripts were treated as DEGs.

### 2.6. Analysis of Putative Genes Involved in Anthraquinone Biosynthesis in the R. yunnanensis Transcriptome

The DEGs found in the *R. yunnanensis* transcriptome were proceeded for GO and KEGG annotation analyses. In combination with the biosynthetic pathways of higher plant anthraquinones reported in the literatures [16,17,18,19,38], we identified 15 putative genes involved in anthraquinone biosynthesis.

### 2.7. RT-qPCR Validation of the Gene Expression Level Quantified Using Illumina Sequencing

To validate the gene expression level quantified using RNA-seq, we selected six of the 15 putative genes, viz., the gene of acetyl-CoA acetyltransferase (*AACT*), the gene of o-succinylbenzoate-CoA ligase (*OSBL*), the gene of 3-hydroxy-3-methylglutaryl-coenzyme A reductase (*HMGR*), the gene of 1-deoxy-D-xylulose-5-phosphate synthase (*DXS*), the gene of isochorismate synthase (*ICS*), and the gene of isopentenyl-diphosphate delta-isomerase (*IPPI*), to analyze the expression levels at different tissues using RT-qPCR.

All RT-qPCR reactions were carried out on StepOnePlus^™^ Real-Time PCR System using AceQ^®^ qPCR SYBR^®^ Green Master (Vazme, Nanjing, China). Each reaction contained 10 μL 2 × AceQ qPCR SYBR Green Master Mix, 0.4 μL of each primer (10 μM), 2 μL of cDNA, and 7.2 μL of ddH_2_O. The RT-qPCR was performed according to the following conditions: denaturation at 95 °C for 5 min, 40 cycles at 95 °C for 10 s, and 60 °C for 30 s. To confirm the specificity of the primers, the melting curve was generated by heating the amplicon from 60 to 95 °C. The products of all reactions were separated via electrophoresis in 1.5% agarose gels. Each PCR reaction was repeated in three biological and technical replicates. The relative quantification of gene expression was computed using the 2^−ΔΔCt^ method [39] and normalized to the expression of *hnRNP* and *TBP*, the two most stable genes in *R. yunnanensis* at the different treatments [40].

### 2.8. RT-qPCR Analysis for 15 Genes Involved in Anthraquinone Biosynthesis

Total RNA was isolated from hairy roots of *R. yunnanensis* using the RNAiso Plus reagent (Takara, Tokyo, Japan), following the manufacturer’s instructions. RNA quality was tested by measuring the A260/280 ratios, and the integrity was accessed by electrophoresis on 1.5% (*w/v*) agarose gels. Only RNA samples with an A260/A280 ratio of 1.9–2.2 were considered as high quality for further analysis. Total RNA samples were reversely transcribed into cDNA with HiScript^®^ II Reverse Transcriptase (Vazyme, Nanjing, China). The cDNA was stored at −80 °C until PCR-based analyses. All RT-qPCR reactions were the same as 2.7. The relative quantification of gene expression was normalized to the expression of *hnRNP*. The sequences of the primers for 15 genes involved in anthraquinone biosynthesis were shown in Supplementary Table S2.

### 2.9. Quantitative Analysis of Six Anthraquinones and Two Naphthoquinones in the Hairy Roots of R. yunnanensis

The quantification of Q3, Q4, Q11, Q12, Q17, Q18, Q19, and Q20 was carried out using UPLC-MS/MS. The analyses were performed using a Waters ACQUITY^®^UPLC H Class system (Waters, Milford, MA, USA) fitted with a Waters XEVO^®^TQD system (Milford, MA, USA) equipped with electrospray ionization (ESI). The conditions for UPLC and MS analyses were performed according to our previous method [8]. The eight compounds in the samples were identified by comparing their retention time and mass spectra with the standards, which were isolated from *Rubia* species in our laboratory. The quantitative data were calculated based on the calibration curves of the individual standards through plotting the MS peak areas versus the corresponding concentrations of each representative standard.

### 2.10. Statistical Analysis

The data in the Tables and Figures were represented as the means ± standard deviations. Statistical analysis was performed through a one-way analysis of variance (ANOVA). Pearson’s correlation coefficients were computed between quantitative variables using SPSS software (version 23.0). Heatmaps were produced in Heatmapper (http://www.heatmapper.ca/expression/, accessed on 25 May 2021).

## 3. Results

### 3.1. Illumina Sequencing and De Novo Sequence Assembly

Total RNA was extracted from roots (R) and a mixture of stems and leaves (SL) (each three replicates), respectively. Sequencing was separately carried out for six samples using the Illumina HiSeq-2500 platform. A total of 34,612,412–41,363,450 clean reads for each sample were obtained from 36,888,628–43,747,160 raw reads by filtering and removing adapters, low quality reads, and unknown nucleotides (*N* > 10%) (Supplementary Table S3). All these data were characterized by Raw Q30 ≥86.46% and Raw GC contents ≥48.18%, respectively. The de novo transcriptome was assembled using Trinity [41] with all the six samples, which generated 636,198 transcripts belonging to 554,646 genes (Supplementary Table S4). These transcripts have a mean length of 300.64 bp and an N50 value of 390 bp. The size distribution of transcripts was shown in Supplementary Figure S2.

### 3.2. Function Annotation Analysis

With the BLASTX, 17.09%, 15.45%, 16.27%, 13.71%, 6.11%, and 11.41% of the 636,198 transcripts were annotated using NR, SwissProt, KEGG, KOG, Pfam, and GO databases, respectively (Table 1). In total, 22.02 percent of transcripts were annotated. The high proportion of annotated transcripts could be attributed to lacking a reference genome of *R. yunnanensis* and strict parameters used in BLASTX. NR annotation revealed that *Coffea canephora*, *Zea mays*, *Hordeum vulgare* subsp. *vulgare*, and *Sesamum indicum* are the top species, which accounted for 28.0%, 6.34%, 3.64%, and 2.72% of the matched transcripts, respectively. We assigned the annotated transcripts to GO categories. GOs were divided into three classes, viz. biological process, cellular component, and molecular function. Within the biological process, metabolic processes were the most highly represented groups (Supplementary Figure S3). According to the KEGG annotation, the three most abundant pathways are signal transduction (7984), carbohydrate metabolism (6549), and global and overview maps (5025) (Supplementary Figure S4).

### 3.3. Identification of Genes Involved in Anthraquinone Biosynthesis

**Figure 1:**
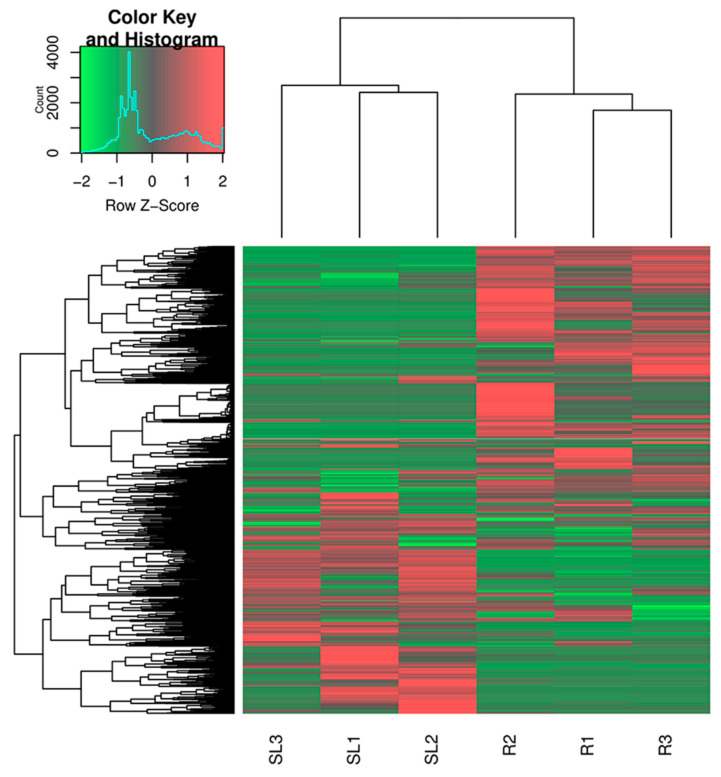
Heat map of gene expression level of the roots (R) samples and a mixture sample of stems and leaves (SL) of *R*. *yunnanensis*.

**Figure 2:**
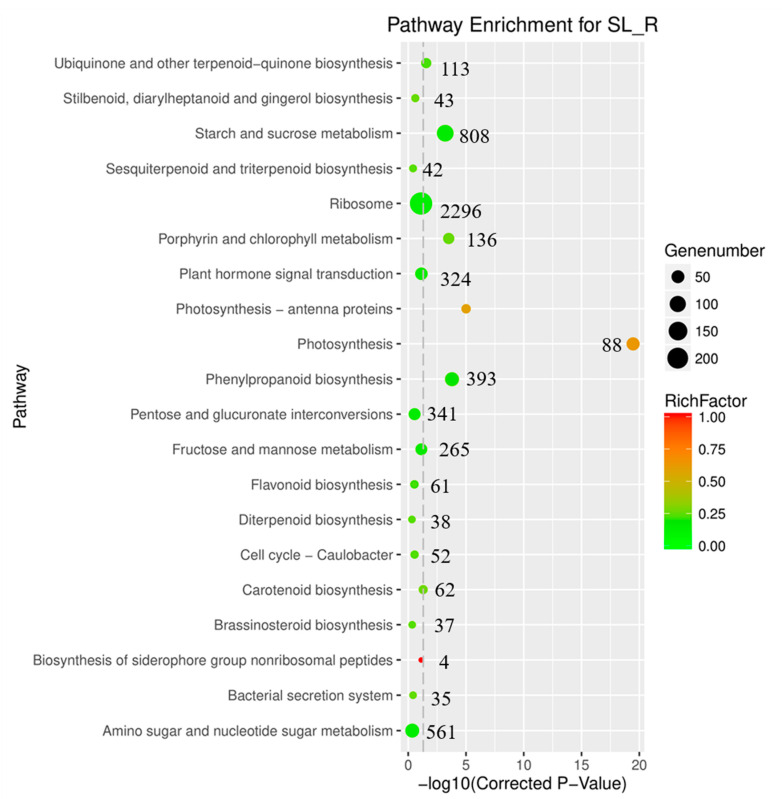
Statistics of DEGs enriched in the KEGG pathway between the samples of roots (R) and a mixture of stems and leaves (SL). Y-axis on the right side shows the number of differentially expressed genes.

**Figure 3:**
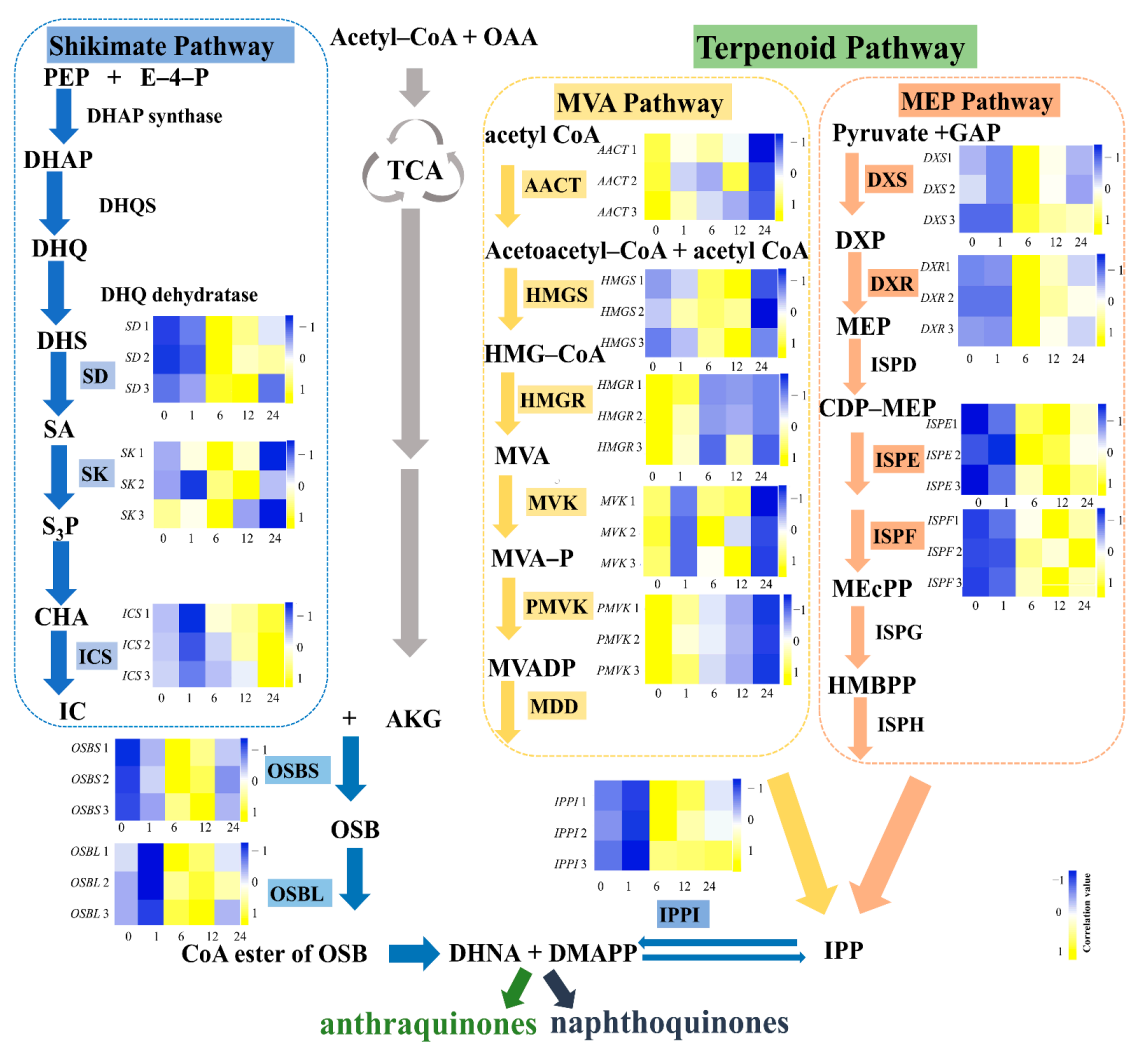
The expression levels of anthraquinone related genes in the hairy roots of *R*. *yunnanensis*. The pathways were re-drawn refering to the literatures [16,17,18,19,38]. Subfigures: the hairy roots were treated by MeJA. 0, 1, 6, 12, and 24 h and each x-axis represents the time points (hours) of each sample; gene expression levels in y-axis were quantified using RT-qPCR; heatmaps were plotted in Heatmapper (http://www.heatmapper.ca/expression/, accessed on 25 May 2021).

According to our previous work, *R. yunnanensis* roots (R) have higher contents of anthraquinones than that of a mixture of stems and leaves (SL) [8]. A heatmap was constructed using a clustering analysis based on the FPKM values, which showed the different gene expression patterns in R and SL samples (Figure 1). We also conducted a differential gene expression analysis between roots and a mixture of stems and leaves using edgeR with FPKM values [37]. A total of 8811 DEGs were identified based on the criteria of FDR < 0.05 and |log_2_Fold change| > 1.5 (Supplementary Figure S5). Among the 8811 genes, 5372 were significantly up-regulated and 3439 were down-regulated in roots. The KEGG enrichment analysis showed that 113 DEGs were involved in the ubiquinone and other terpenoid-quinone pathways (Figure 2). According to the NR annotation and literatures [16,17,18,19,38], 15 of 113 DEGs encoded anthraquinone biosynthetic enzymes, viz., isochorismate synthase (ICS), acetyl-CoA acetyltransferase (AACT), shikimate kinase (SK), 3-hydroxy-3-methylglutaryl coenzyme A synthase (HMGS), o-succinylbenzoate synthase (OSBS), o-succinylbenzoate-CoA ligase (OSBL), isopentenyl-diphosphate delta-isomerase (IPPI), 1-deoxy-D-xylulose-5-phosphate synthase (DXS), 1-deoxy-D-xylulose 5-phosphate reductoisomerase (DXR), 3-hydroxy-3-methylglutaryl-coenzyme A reductase (HMGR), mevalonate kinase (MVK), shikimate dehydrogenase (SD), phosphomevalonate kinase (PMVK), 4-diphosphocytidyl-2C-methyl-D-erythritol kinase (ISPE), and 2-C-methyl-D-erythritol 2,4-cyclodiphosphate synthase (ISPF) (Figure 3 and Supplementary Table S5). Among the 15 genes, the expression of *IPPI*, *ICS*, *SD*, *OSBL*, *HMGR*, *DXR*, *DXS*, *PMVK*, *OSBS*, and *AACT* was higher in roots, while the expression of *SK, MVK, ISPF*, *ISPE*, and *HMGS* was the opposite.

### 3.4. Validation of the Gene Expression Level

To validate the gene expression level quantified by RNA-seq, the expressions of *ICS*, *AACT*, *DXS*, *IPPI*, *OSBL*, and *HMGR* were quantified using RT-qPCR. The results indicated that the expression level quantified using RT-qPCR was consistent with RNA-seq (Supplementary Figure S6). Both of them showed that the expression levels of the six genes in the roots (R) were higher than that in a mixture of stems and leaves (SL).

### 3.5. Gene Expressions Levels and Anthraquinone Contents in the Hairy Roots of R. yunnanensis under MeJA Treatment

To gain further insights into anthraquinone biosynthesis, the hairy roots of *R. yunnanensis* were treated by MeJA for 1, 6, 12, and 24 h, respectively. Then, the expression levels of the 15 putative genes involved in anthraquinone biosynthesis and the contents of six anthraquinones and two naphthoquinones were measured using RT-qPCR and UPLC-MS/MS, respectively. All treatments were compared with the control (0 h).

**Figure 4:**
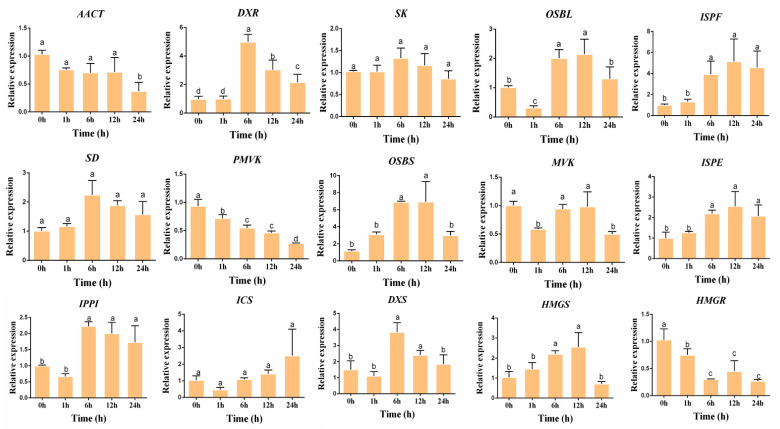
The gene expression in the hairy roots of *R. yunnanensis* after MeJA treatment for 1, 6, 12, and 24 h. *hnRNP* was used as the reference gene. The vertical axis represents the relative gene expression level. Different lowercase letters for the same metabolite indicate significant differences (*p* < 0.05) between the samples.

The results showed that the expressions for nine of these 15 genes was up-regulated after 6 and 12 h of MeJA treatment but was down-regulated after 24 h. The expression levels of *HMGS*, *OSBS*, *OSBL*, *ISPE*, *ISPF*, *IPPI*, *SD*, *DXS*, and *DXR* were up-regulated after being treated for 6 or 12 h, with the fold changes of 2.45, 5.96, 2.56, 2.12, 5.12, 2.23, 2.22, 2.55, and 5.12, respectively. The expression level of *ICS* increased 2.42 times after MeJA treatment for 24 h. However, *AACT*, *MVK*, *SK, HMGR*, and *PMVK* significantly reduced by 64.0%, 50.2%, 16.1%, 73.8%, and 70.3% at 24 h, respectively (Figure 4).

Eight quinones were found in *R. yunnanensis*, including two anthraquinones aglycones (Q3 and Q4), four anthraquinones glycosides (Q12, Q20, Q19, and Q11), and two naphthoquinones (Q17 and Q18). The contents of these anthraquinones were determined by UPLC-MS/MS analysis. The results showed the contents of Q4 increased by 28.01% after MeJA treatment for 24 h. The contents of Q3 increased by 23.05% at 6 h. However, the contents of Q11, Q20, Q19, and Q12 significantly decreased by 16.23%, 54.33%, 33.01%, and 33.30% for 6, 24, 12, and 24 h. The contents of Q17 and Q18 contents showed no significant difference at 0 or 24 h (Supplementary Figure S7).

### 3.6. Correlation Analysis between the Expression Levels of Genes Involved in Anthraquinone Biosynthesis and Anthraquinone Contents

**Figure 5:**
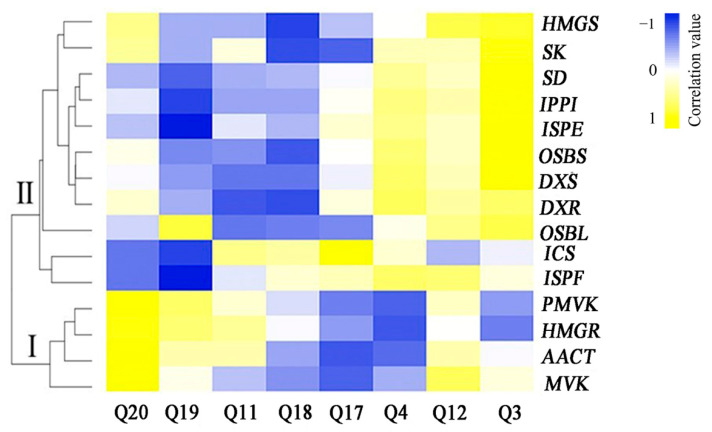
Pearson correlation for the expression levels of 15 genes and the contents of six anthraquinones and two naphthoquinones. Heatmaps were generated using Heatmapper (http://www.heatmapper.ca/expression/, accessed on 25 May 2021).

We treated the hairy roots of *R. yunnanensis* with MeJA and compared the expression levels of 15 genes (*ICS*, *AACT*, *SK*, *HMGS*, *OSBS*, *OSBL*, *IPPI*, *DXS*, *DXR*, *HMGR*, *MVK*, *SD*, *PMVK*, *ISPE*, and *ISPF*), the contents of six anthraquinone (Q3, Q4, Q12, Q20, Q19, and Q11), and two naphthoquinones (Q17 and Q18). Our results revealed that ten genes were upregulated and five were downregulated (Figure 4). Two anthraquinones were significantly increased. Four anthraquinones and two naphthoquinones were decreased after MeJA treatment (Supplementary Figure S7). To study the expression pattern of these 15 putative genes, we performed a correlation analysis between the gene expression levels and anthraquinone contents. As shown in Figure 5, 15 genes were grouped into two clusters based on the correlation values. The the contents of Q20, Q19, and Q12 were positively correlated with the expression of genes involved in the MVA pathways, which were positioned in Cluster I, including *MVK*, *AACT*, *PMVK*, and *HMGR*.

**Figure 6:**
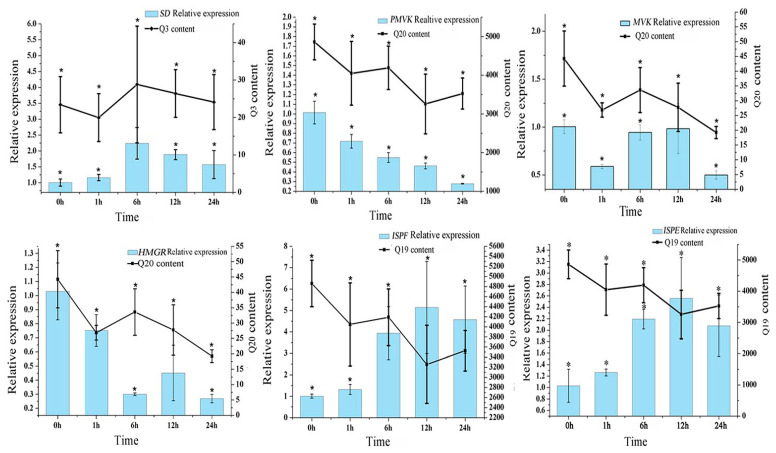
The expression levels of six genes were significantly related to the contents of three anthraquinones after 0, 1, 6, 12, and 24 h under MeJA treatment. “*” indicates significant differences (*p* < 0.05) between the samples.

We used a |correlation coefficient| > 0.5 and *p* < 0.05 as the cutoff for a significant correlation (Supplementary Table S6). The results showed a significant correlation between three anthraquinones and six genes. The expressions of *HMGR*, *MVK*, and *PMVK* were positively correlated with the Q20 contents, with the correlation coefficients of 0.648, 0.694, and 0.797, respectively. The expression of *SD* was positively correlated with the Q3 contents. There was a negative correlation between the expression levels of *ISPE* and *ISPF* genes and the Q19 contents, with the correlation coefficients of −0.671 and −0.721, respectively (Figure 6).

## 4. Discussion

Due to its significant efficacy, *R*. *yunnanensis* is commonly used in promoting blood circulation, stretching tendons, and eliminating blood stasis. *R. yunnanensis* is rich in anthraquinones with some pharmacological effects [4,5,6,7]. Abiotic stress could promote the accumulation of anthraquinones in hairy roots [42,43,44]. For instance, elicitation with 200 µM MeJA increased the anthraquinones in hairy roots of *Oplopanax elatus* by 2.0-fold [45]. These findings indicated that MeJA can be used in hairy culture systems to increase metabolites production. In this study, when using a concentration of 200 µM MeJA, the anthraquinone contents increased 35.11%. It supported the opinion that effective elicitors, such as MeJA, were able to improve the accumulation of anthraquinones in the hairy roots of *R*. *yunnanensis* [31].

Anthraquinones in *Rubia* could be biosynthesized through the combination of shikimate and MVA/MEP pathways. Several genes in biosynthetic pathways of anthraquinones have been identified from the genus *Rubia*, including *DXS*, *DXR*, *ICS*, *OSBS*, *OSBL*, and *IPPI* [46,47,48]. Here, we reported the first de novo transcriptome of *R. yunnanensis* using the Illumina HiSeq platform. Most of the genes encoding enzymes, which have been reported to be involved in the biosynthesis of the anthraquinones [17,18,19], were present in the transcriptome of *R*. *yunnanensis*. Fifteen transcripts were found in the MEP pathway (*DXS*, *DXR*, *ISPF*, *ISPF*, and *IPPI*), MVA pathway (*HMGR*, *AACT*, *HMGS*, *MVK*, and *PMVK*), and shikimate pathway (*SD, SK*, *ICS, OSBS*, and *OSBL*), respectively. In addition, the correlation analysis in Figure 6, showed that the expressions of *HMGR*, *MVK*, *PMVK,* and *SD* were positively correlated with the anthraquinone contents. These genes were involved in the MVA (*HMGR*, *MVK*, and *PMVK*) and shikimate (*SD*) pathways. *HMGR* and *MVK* are rate-limiting enzyme genes [49,50] and participate in a series of biochemical reactions, such as anthraquinones, triterpenes, and phytosterols synthesis. *PMVK* is found to catalyze a key step in isoprenoid biosynthesis, converting mevalonate 5-phosphate to mevalonate 5-diphosphate in the MVA pathway [51,52]. *SD* was found to catalyze shikimate synthesis, representing the fourth step of the shikimate pathway, a conserved biosynthetic route commonly in plants, fungi, and bacteria [53,54]. It was also reported that the expressions of shikimate pathway genes responded to various types of elicitors [55]. Our results also showed that the expression level of *SD* was up-regulated at 6 h, possibly responding to the MeJA elicitor.

In addition, we found the contents of anthraquinones were increased after MeJA treatment, while two naphthoquinones (Q17 and Q18) showed no significant difference. These results were consistent with the cell suspension of *Rubia cordifolia* [56]. The biosynthetic pathways of anthraquinones and naphthoquinones are both derived from 1, 4-dihydroxy-2-naphthoic acid (DHNA) synthesized in the shikimate pathway, but branching occurs after the prenylation with an isopentenyl moiety IPP [18] (Figure 3). The IPP origin was synthesized from the MVA and MEP pathways. In our study, the genes in the MVA (*HMGR*, *MVK*, and *PMVK*) positively correlated with the anthraquinone contents, which may imply that MeJA mainly regulated the MVA pathway through these three genes to synthesize IPP for the accumulation of anthraquinones. However, the synthesis of IPP of naphthoquinone (Q17) appeared to be mainly from the MEP pathway [56]. Moreover, the function of the genes of the MEP pathway is influenced by the light [57,58], while the MVA pathway is negatively regulated by light [59]. In our study, the hairy root culture of *R. yunnanensis* was in darkness, which may promote the regulation of the MVA pathway leading to the increase of anthraquinones. These findings implied MVA pathway may play an important role in the biosynthesis of anthraquinones in *R. yunnanensis*, especially the four genes *HMGR*, *MVK*, *PMVK*, and *SD*, which could contribute to the production of anthraquinones. However, the integration and regulation of four targeted genes may need further investigation by using CRISPR activation/interference [60,61].

In the late stage of anthraquinone biosynthesis, the backbone of anthraquinones experiences various modifications, such as SAM-dependent methyltransferases [62], Cytochrome P450 (CYPs) [63], and UDP-glucose glucosyltransferases (UDPGs). These modification enzymes play different roles in anthraquinones’ metabolism. For example, CYPs could catalyze most of the oxidative reactions, including epoxidation, dealkylation, dehydration, and carbon–carbon bond cleavage of anthraquinones [64]. UDPGs catalyzed glycosylation at the site of the hydroxyl group to produce glycosylated anthraquinones [20]. In this study, 18 and 2 transcripts were identified for the first time as *CYPs* and *UDPGs*, respectively, from the *R. yunnanensis* transcriptome, which could contribute to a better understanding of the anthraquinone biosynthesis.

## 5. Conclusions

*R. yunnanensis* is rich in bioactive anthraquinones, while little is known regarding its genes in the anthraquinone biosynthesis. In this study, we reported the first de novo assembled transcriptome and identified 15 putative genes involved in the anthraquinone biosynthesis pathway in *R. yunnanensis*. Four of these genes (*HMGR*, *MVK*, *PMVK,* and *SD*) exhibited a high positive correlation in the anthraquinone contents. This study has a potential to discover putative genes in anthraquinone biosynthesis pathways in the *Rubia* plants.

## Figures and Tables

**Table 1:** Statistical results of transcript annotation.

Database	Number of Annotated Transcripts	Ratio of Annotated Transcripts (%)	Number of Annotated Genes	Ratio of Annotated Genes (%)
All assembly transcripts	636,198	NA	NA	NA
All assembly genes	554,646	NA	NA	NA
NR	108,753	17.09	77,188	13.92
Swiss-Prot	98,311	15.45	76,703	13.83
KEGG	103,529	16.27	73,112	13.18
KOG	87,215	13.71	67,466	12.16
Pfam	38,877	6.11	29,369	5.30
GO	72,573	11.41	51,102	9.21
All annotated transcripts	140,078	22.02	107,631	19.41

## Data Availability

Assembled transcriptomes, transcript annotation, and the differential expressed transcripts, and annotation of DEGs are available at the figshare: https://doi.org/10.6084/m9.figshare.17711582.v1, accessed on 2 January 2022.

## Supplementary Materials

The following supporting information can be downloaded at: https://www.mdpi.com/article/10.3390/genes13030521/s1, Figure S1: Chemical structures of six anthraquinones and two naphthoquinones isolated from *R. yunnanensis*, Figure S2: Length distribution of the assembled *R. yunnanensis* transcripts, Figure S3: GO classification of *R. yunnanensis* assembled transcripts, Figure S4: KEGG classification of assembled transcripts from *R. yunnanensis*, Figure S5: Number of differentially expressed genes (DEGs) in the samples between roots (R) and a mixture of stems and leaves (SL) of *R. yunnanensis*, Figure S6: RT-qPCR validation of the expression levels of six putative key genes involved in anthraquinone biosynthesis pathways [40], Figure S7: Contents of anthraquinones and naphthoquinones in the hairy roots of *R. yunnanensis* after 1, 6, 12, and 24 h’s MeJA treatment, Table S1: RNA in roots (R), a mixture of stems and leaves (SL) of *R. yunnanensis*, Table S2. The sequences of the primers for 15 genes involved in anthraquinone biosynthesis, Table S3: A summary of raw data, Table S4: Statistics of transcript assembly, Table S5: Transcripts associated with ubiquinone and terpenoid-quinone biosynthesis according to the KEGG pathway mapping, Table S6: Correlation values between 15 genes involved in the anthraquinone biosynthesis and the contents of six anthraquinones and two naphthoquinones.
