# Supplementary material for: De Novo Transcriptome Analysis Reveals Putative Genes Involved in Anthraquinone Biosynthesis in Rubia yunnanensis
Source: Genes (Basel). 2022 Mar 16;13(3):521. doi: 10.3390/genes13030521 (PMC8954821; doi:10.3390/genes13030521)
Supplement: Supplementary file 1 [file genes-13-00521-s001.zip › genes-1588501-supplementary.pdf]

DE Novo Transcriptome Analysis Reveals Putative Genes Involved in Anthraquinone Biosynthesis in *Rubia yunnanensis*

Rongfei Zhang [1], Yuanyuan Miao [1], Lingyun Chen [1], Shanyong Yi [1,2,*] and Ninghua Tan [1,*]

[1] Department of TCMs Pharmaceuticals, School of Traditional Chinese Pharmacy, China Pharmaceutical University, Nanjing 211198, China; zrf1327182884@126.com (R.Z.); miaoyuanyuan1989@163.com (Y.M.); lychen@cpu.edu.cn (L.C.)
[2] Department of Biological and Pharmaceutical Engineering, West Anhui University, Lu'an 237012, China
* Correspondence: 02000157@wxc.edu.cn (S.Y.); nhtan@cpu.edu.cn (N.T.)

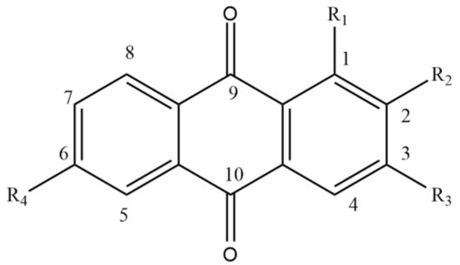

Name	R_1	R_2	R_3	R_4
2-hydroxymethyl-1,3-dihydroxy-AQ-3-O-*β*-D-xylopyranosyl-(1→6)-*β*-D-glucopyranoside **Q12**	OH	CH_2OH	OGlc(6→1)Xyl	H
3-hydroxy-2-hydroxymethyl-AQ **Q4**	H	CH_2OH	OH	H
rubiquinone-3-O-*β*-D-xylopranosyl-(1→6)-*β*-D-glucopyranoside **Q20**	OH	CH_3	OGlc(6→1)Xyl	OH
rubiquinone-3-O-(4'-O-acetyl)-*α*-L-rhamnopyranosyl)-(1→2)-*β*-D-glucopyranoside **Q19**	OH	CH_3	(4'-O-Ac)-OGlc(2→1)Rha	OH
1-hydroxy-2-hydroxymethylene-AQ-11-O-*β*-D-glucopyranosyl-(1→6)-*β*-D-glucopyranoside **Q11**	OH	$CH_2OGlc(6\rightarrow1)Glc$	H	H
rubiquinone **Q3**	OH	CH_3	OH	OH

AQ: 9,10-anthraquinone

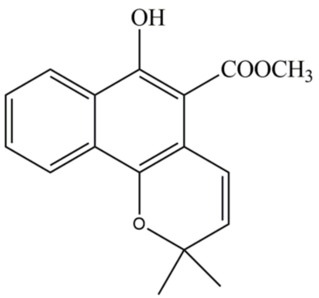

Q17

Mollugin

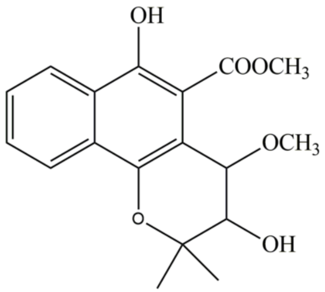

Q18

1'-methoxy-2'-hydroxy-9,10-dihydromullugin

Supplementary Figure S1. Chemical structures of six anthraquinones and two naphthoquinones isolated from *R. yunnanensis*.

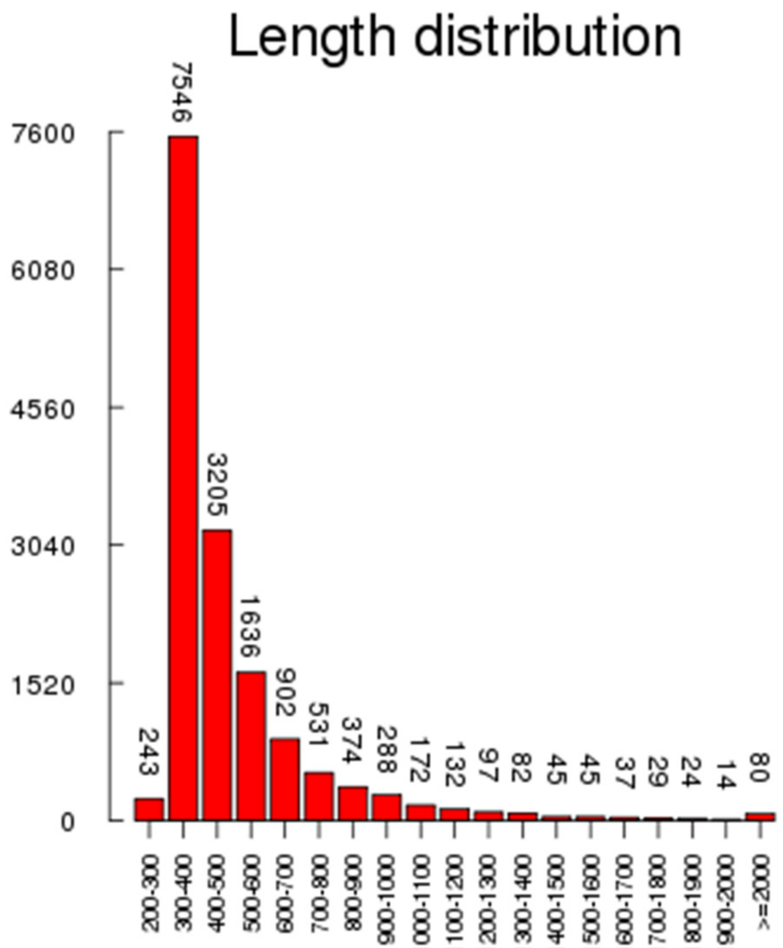

Supplementary Figure S2. Length distribution of the assembled *R. yunnanensis* transcripts.

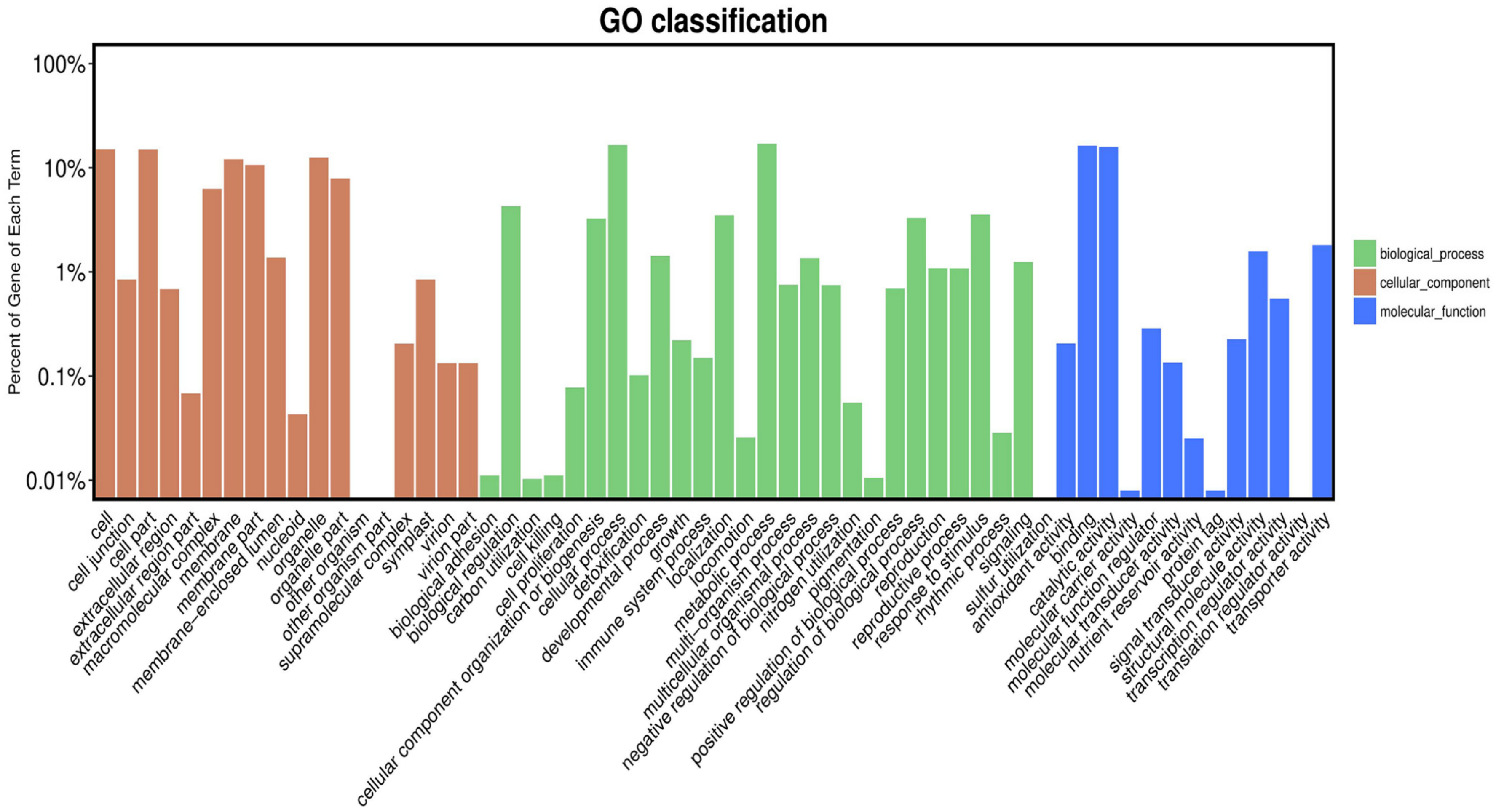

Supplementary Figure S3. GO classification of *R. yunnanensis* assembled transcripts.

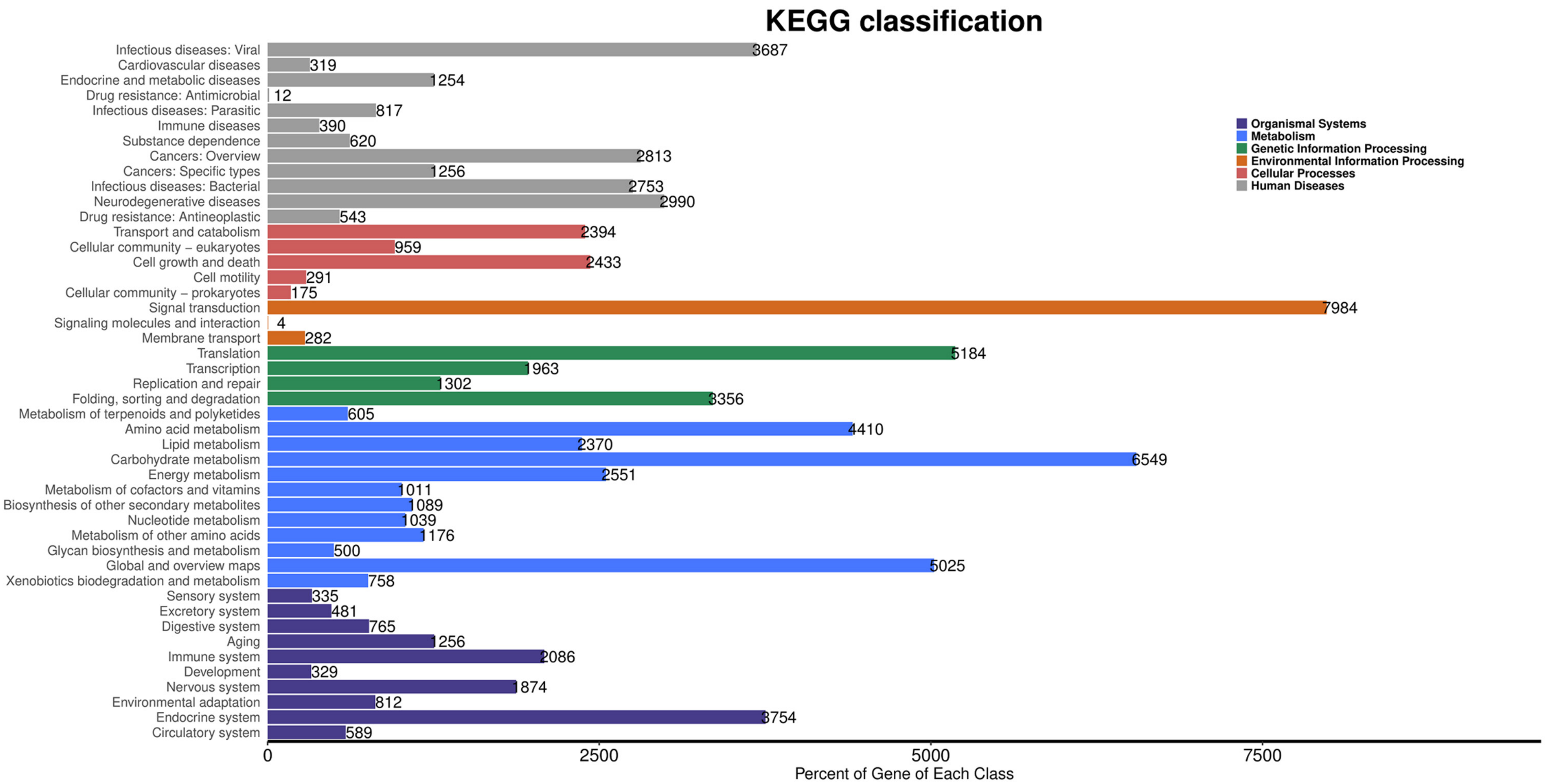

Supplementary Figure S4. KEGG classification of assembled transcripts from *R. yunnanensis*.

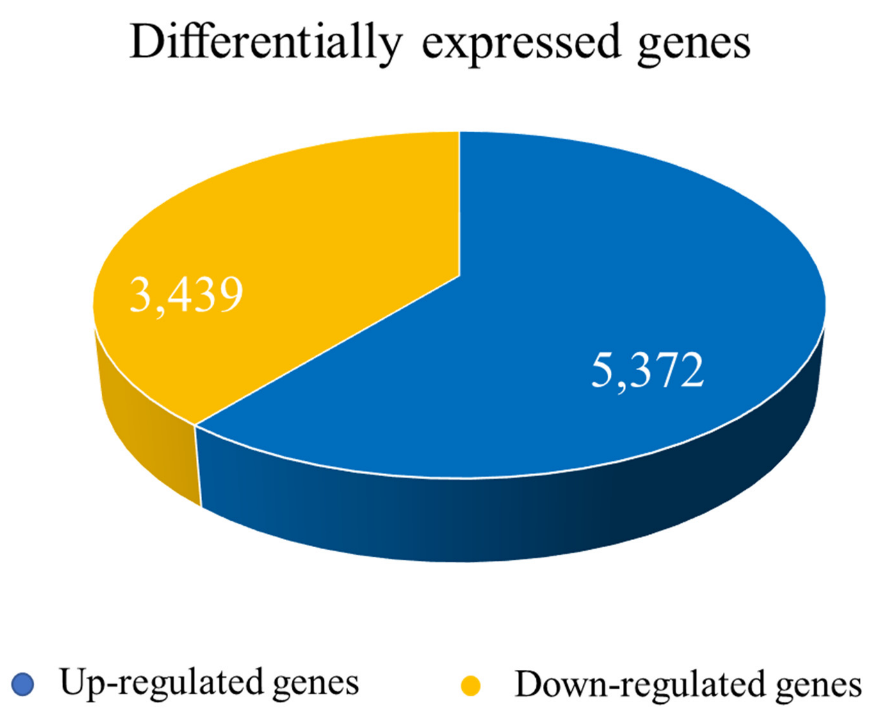

Supplementary Figure S5. Number of differentially expressed genes (DEGs) in the samples between roots (R) and a mixture of stems and leaves (SL) of *R. yunnanensis*.

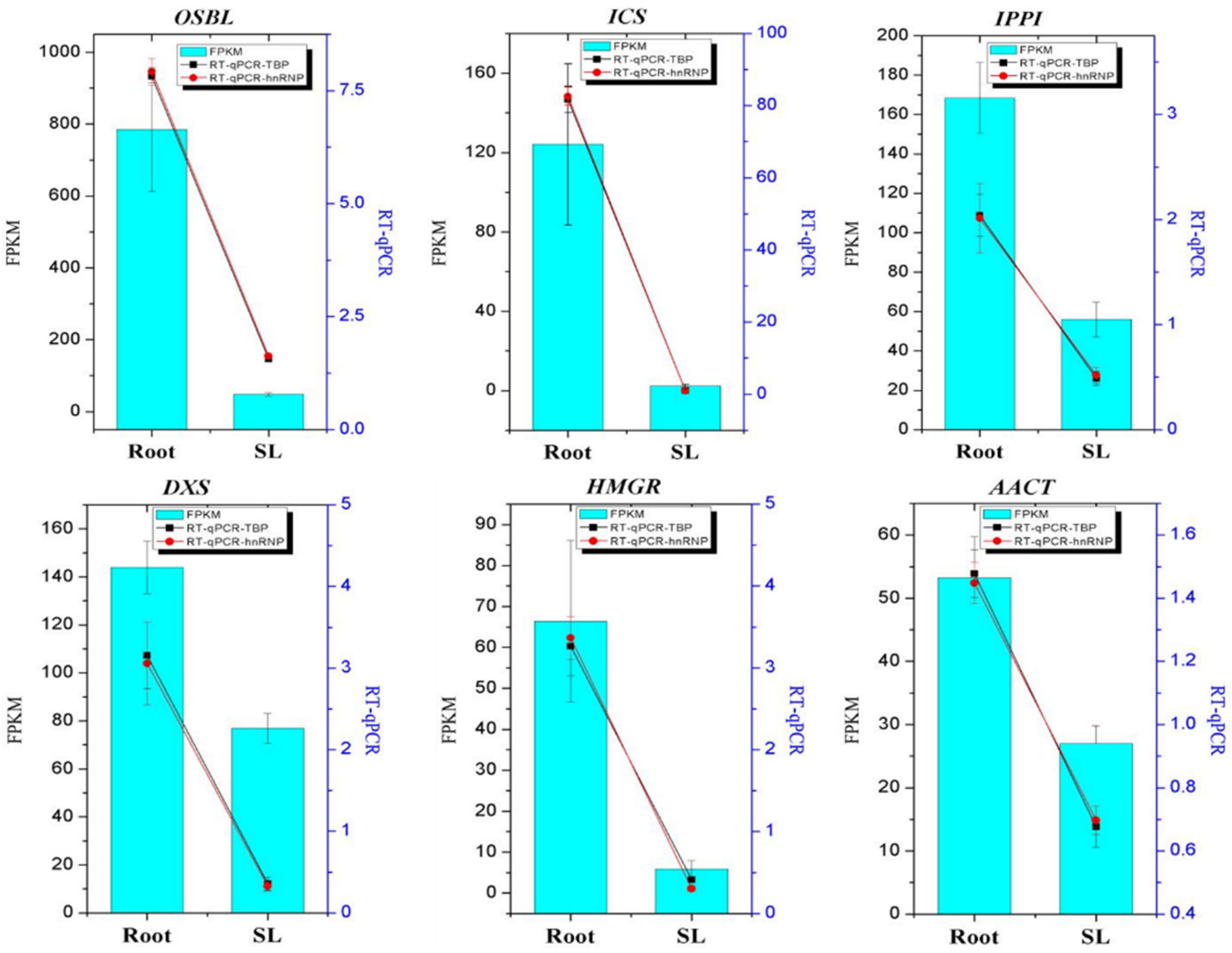

Supplementary Figure S6. RT-qPCR validation of the expression levels of six putative key genes involved in anthraquinone biosynthesis pathways. *hnRNP* and *TBP* were used as reference genes. Columns indicate relative expression levels of six genes measured by FPKM (left y-axis).［40］

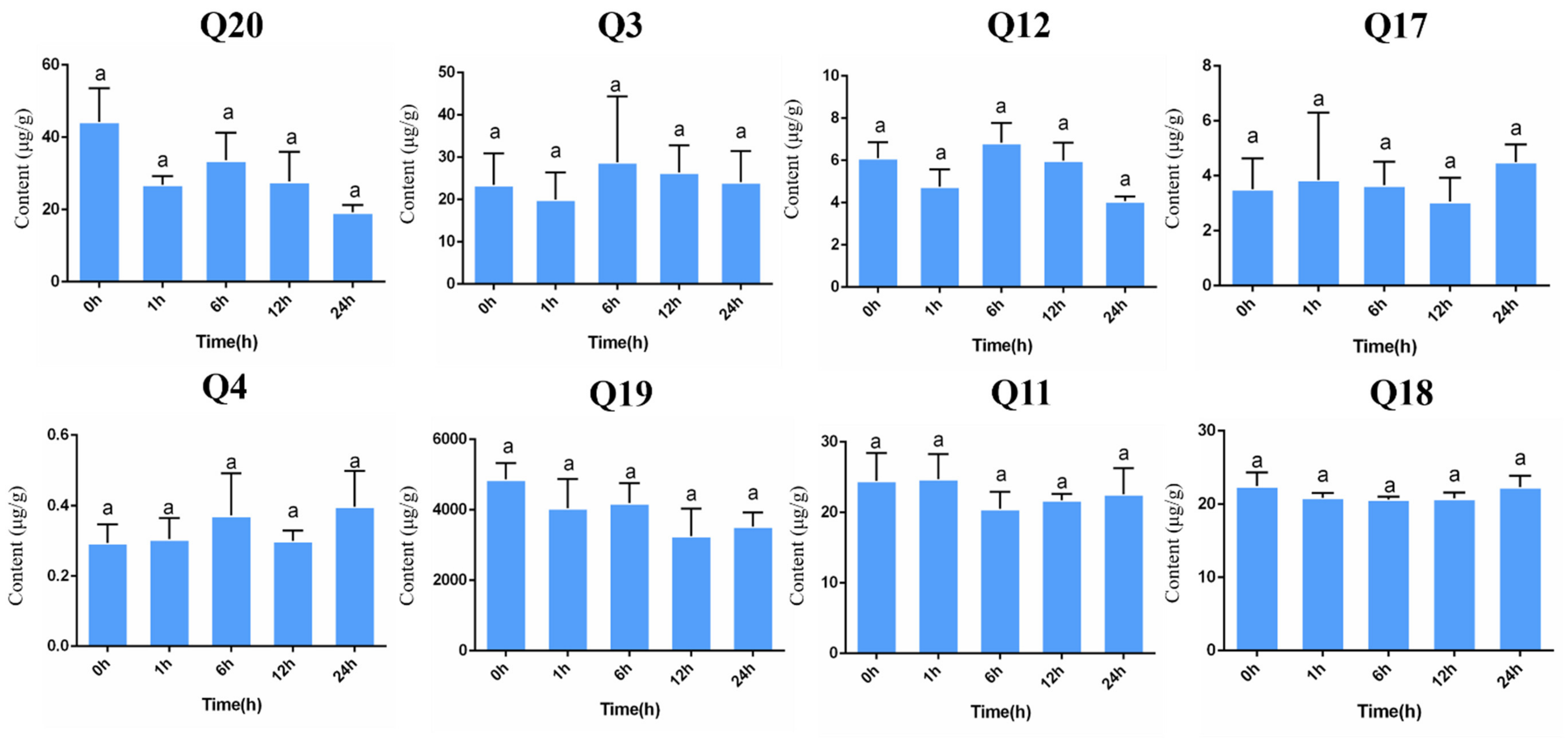

Supplementary Figure. S7. Contents of anthraquinones and naphthoquinones in the hairy roots of *R. yunnanensis* after 1, 6, 12, and 24 h' s MeJA treatment. Y-axis represents contents (μg/g) and X-axis represents MeJA inducing time.

Supplementary Table S1. RNA in roots (R), stems and leaves (SL) of *R. yunnanensis*.

Sample	Concentration (ng/μL)	Total amount (μg)	OD260/280	RIN	Volume (μL)
R1 RNA	340	6.8	2.126	8.3	20
R2 RNA	236	4.7	2.120	8.2	20
R3 RNA	171	3.4	2.130	8.0	20
SL1 RNA	163	3.3	2.104	6.9	20
SL2 RNA	225	4.5	2.146	6.6	20
SL3 RNA	235	4.7	2.128	6.8	20

RIN = RNA integrity number.

Supplementary Table S2. The sequences of the primers for 15 genes involved in anthraquinone biosynthesis.

Genes	Primer sequence (5′-3′)	Primers Tm(°C)
OSBS	F: GGTACTTACGTCATTTTCTTGTCCC<br>R: TGAATATCAGCTACCAGTGGACTTG	57.9/57.9
ICS	F: CATCCCTTCATCCAACTCCAG<br>R: GCTTCCTTCTACCACGCCA	57.6/57.3
HMGR	F: TTGAGGTCGGGACAGTAGGTG<br>R: CAGCAGCCGAGCATTTGA	59.5/54.9
DXS	F: TTCTCTGCCTACGGCTACTCTT<br>R: AACTTTTCGCTGCCTCGC	57.7/54.9
SD	F: AGACACTCATTTGTGCCCCATT<br>R: CACCTTCATACTGACCACCCTC	55.8/59.5
IPPI	F: AACCGAGACGAGTTGAGGGA<br>R: ATGTCAATAGCATCAGTCAGGGT	57.4/56
ISPE	F: AGTTTTCGCTATCGCCTTCG<br>R: CATTACTGCTGCCACCACCT	55.4/57.4
PMVK	F: CAGTGGTGGCTTCTGCTCCT<br>R: CCAAGACCACGCCCAAGTA	59.5/57.3
SK	F: AATGATGGGCTCTGGCAAA<br>R: TAGTTCCTCCAACCCCTTCC	53/57.4
DXR	F: GCCTGTTGAGAAGTTGAAAGAAG<br>R: GATGATGGATTGGGGGTGA	56/55.2
HMGS	F: TTTGCCCGATTGGTGTTCA<br>R: CAAGGTCACGGCTTTGGTAG	53/57.4
MVK	F: CGTGTTGACGCTGCTACCTAC<br>R: TGCTTGAAAATGTGGGATGG	59.5/53.4
ISPF	F: AACAGAACGACGACGCATACC<br>R: GGCTGCTGAGACAAACGGA	57.6/57.3
AACT	F: GCTGTATCACTGGGGCATCC<br>R: CAAACACCTCCAACGCCTACT	59.5/57.6
OSBL	F: TGCTGGCTACACTGAGGATGA<br>R: CCTTGACCGCTGCTTGAACT	57.6/57.4

Supplementary Table S3. A summary of raw data. Roots (R1, R2 and R3), and a mixture of stems and leaves (SL1, SL2 and SL3) of *R. yunnanensis*.

Sample	Read Len.	Raw Reads	Clean Reads	Raw Base (G)	Clean Base (G)	Raw Q20 (%)	Clean Q20 (%)	Raw Q30 (%)	Clean Q30 (%)	Raw GC (%)	Clean GC (%)	Adapter (%)
R1	150	43,202,582	41,132,788	6.48	6.17	94.16	95.09	87.87	89.18	50.54	50.77	1.54
R2	150	36,888,628	34,612,412	5.53	5.19	93.19	94.7	86.46	88.5	50.04	50.32	2.03
R3	150	42,808,828	41,342,472	6.42	6.2	94.73	95.42	88.72	89.72	50.13	50.14	1.36
SL1	150	43,110,678	41,096,062	6.47	6.16	94.17	95.1	87.91	89.21	49.71	49.9	1.48
SL2	150	43,747,160	41,363,450	6.56	6.2	93.81	94.99	87.47	89.08	48.18	48.36	1.95
SL3	150	42,661,982	40,671,204	6.4	6.1	94.16	95.12	87.87	89.23	49.03	49.17	1.42

Supplementary Table S4. Statistics of transcript assembly.

Item	Value
Total number sequences	636,198
Total number of genes	554,646
Total base of sequences (Mb)	191
Maximum sequence length (bp)	11,222
Minimum sequence length (bp)	81
Average sequence length (bp)	300.64
Median contig length (bp)	150
N50 (bp)	390
Percent GC (%)	43.55

Supplementary Table S5. Transcripts associated with ubiquinone and terpenoid-quinone biosynthesis according to the KEGG pathway mapping.

Pathway	Enzyme name	EC number	Gene	Unigene
Shikimate pathway	shikimate dehydrogenase	1.1.1.25	*SD*	TRINITY_DN43879_c0_g2
	shikimate kinase	2.7.1.71	*SK*	TRINITY_DN107580_c0_g2
	isochorismate synthase	5.4.99.6	*ICS*	TRINITY_DN125672_c2_g2
	o-succinylbenzoate-CoA ligase	6.2.1.26	*OSBL*	TRINITY_DN125216_c2_g1
Terpenoid pathway	3-hydroxy-3-methylglutaryl-coenzyme A reductase	1.1.1.34	*HMGR*	TRINITY_DN129754_c4_g1
	1-deoxy-D-xylulose-5-phosphate reductoisomerase	1.1.1.267	*DXR*	TRINITY_DN129547_c2_g3
	mevalonate kinase	2.7.1.36	*MVK*	TRINITY_DN130215_c1_g2
	phosphomevalonate kinase	2.7.4.2	*PMVK*	TRINITY_DN28921_c0_g1
	isopentenyl-diphosphate delta-isomerase	5.3.3.2	*IPPI*	TRINITY_DN131965_c2_g1
	acetyl-CoA acetyltransferase	2.3.1.9	*AACT*	TRINITY_DN302889_c0_g1
	1-deoxy-D-xylulose-5-phosphate synthase	2.2.1.7	*DXS*	TRINITY_DN124410_c3_g1
	2-C-methyl-D-erythritol 2,4-cyclodiphosphate synthase	4.6.1.12	*ISPF*	TRINITY_DN122309_c0_g1
	4-diphosphocytidyl-2C-methyl-D-erythritol kinase	2.7.1.148	*ISPE*	TRINITY_DN126438_c0_g1
	3-hydroxy-3-methylglutaryl coenzyme A synthase	2.3.3.10	*HMGS*	TRINITY_DN131039_c3_g1
	o-succinylbenzoate synthase	4.2.1.113	*OSBS*	TRINITY_DN319173_c0_g1

Supplementary Table S6. Correlation values between 15 genes involved in the anthraquinone biosynthesis and the contents of six anthraquinones and two naphthoquinones.

Metabolites	Q20		Q18		Q19		Q4		Q12		Q11		Q3		Q17	
**Gene**	**Pearson**	**P**	**Pearson**	**P**	**Pearson**	**P**	**Pearson**	**P**	**Pearson**	**P**	**Pearson**	**P**	**Pearson**	**P**	**Pearson**	**P**
IPPI	-0.129	0.647	-0.26	0.349	-0.459	0.085	0.140	0.618	0.077	0.786	-0.254	0.361	0.33	0.230	-0.057	0.841
ISPF	-0.525	0.044	-0.12	0.670	-0.721**	0.002	0.09	0.749	0.071	0.801	-0.266	0.337	-0.133	0.036	-0.058	0.862
ISPE	-0.288	0.298	-0.322	0.241	-0.671*	0.006	0.088	0.755	-0.0108	0.703	-0.211	0.45	0.310	0.260	-0.049	0.714
SD	-0.259	0.352	-0.265	0.341	-0.471	0.072	0.149	0.596	0.047	0.867	-0.273	0.325	0.502*	0.045	-0.1	0.724
SK	0.206	0.461	-0.458	0.086	-0.205	0.463	0.149	0.597	0.139	0.621	0.074	0.795	0.424	0.116	-0.412	0.127
HMGS	0.115	0.682	-0.501	0.057	-0.277	0.318	-0.092	0.744	0.219	0.433	-0.274	0.324	0.261	0.347	-0.238	0.392
HMGR	0.648*	0.009	0.142	0.615	0.493	0.062	-0.307	0.266	0.169	0.547	0.404	0.135	-0.219	0.433	-0.135	0.632
ICS	-0.322	0.242	0.079	0.781	-0.403	0.137	0.017	0.951	-0.203	0.467	0.129	0.646	-0.083	0.768	0.278	0.315
AACT	0.679	0.005	-0.115	0.684	0.324	0.239	-0.256	0.358	0.338	0.218	0.337	0.219	0.119	0.672	-0.310	0.260
MVK	0.694**	0.004	-0.237	0.395	0.167	0.551	-0.144	0.608	0.507	0.054	-0.095	0.735	0.191	0.496	-0.384	0.157
PMVK	0.797*	0.003	0.10	0.724	0.615*	0.015	-0.29	0.294	0.362	0.184	0..328	0.233	-0.133	0..636	-0.217	0.437
DXS	0.002	0.995	-0.343	0.21	-0.187	0.505	0.157	0.576	0.063	0.824	-0.329	0.184	0.140	0.618	-0.009	0.975
DXR	-0.064	0.822	-0.348	0.204	-0.266	0.328	0.245	0.378	0.094	0.738	-0.352	0.198	0.398	0.142	-0.088	0.756
OSBL	-0.054	0.849	-0.251	0.355	0.427	0.113	0.121	0.668	0.293	0.288	-0.274	0.323	0.4	0.139	-0.228	0.414
OSBS	-0.096	0.734	-0.446	0.096	-0.363	0.184	0.092	0.745	-0.035	0.902	-0.340	0.215	0.238	0.382	-0.124	0.660
